# Identification of *PLCL1* Gene for Hip Bone Size Variation in Females in a Genome-Wide Association Study

**DOI:** 10.1371/journal.pone.0003160

**Published:** 2008-09-08

**Authors:** Yao-Zhong Liu, Scott G. Wilson, Liang Wang, Xiao-Gang Liu, Yan-Fang Guo, Jian Li, Han Yan, Panos Deloukas, Nicole Soranzo, Usha Chinnapen-Horsley, Alesandra Cervino, Frances M. Williams, Dong-Hai Xiong, Yin-Ping Zhang, Tian-Bo Jin, Shawn Levy, Christopher J. Papasian, Betty M. Drees, James J. Hamilton, Robert R. Recker, Tim D. Spector, Hong-Wen Deng

**Affiliations:** 1 School of Medicine, University of Missouri - Kansas City, Kansas City, Missouri, United States of America; 2 School of Medicine and Pharmacology, The University of Western Australia and Department of Endocrinology and Diabetes, Sir Charles Gairder Hospital, Nedlands, Western Australia; 3 Twin Research and Genetic Epidemiology Unit, St Thomas' Hospital, King's College London, London, United Kingdom; 4 The Key Laboratory of Biomedical Information Engineering of Ministry of Education and Institute of Molecular Genetics, School of Life Science and Technology, Xi'an Jiaotong University, Xi'an, People's Republic of China; 5 Wellcome Trust Sanger Institute, Wellcome Trust Genome Campus, Hinxton, United Kingdom; 6 Osteoporosis Research Center, Creighton University, Omaha, Nebraska, United States of America; 7 Vanderbilt Microarray Shared Resource, Vanderbilt University, Nashville, Tennessee, United States of America; 8 Laboratory of Molecular and Statistical Genetics, College of Life Sciences, Hunan Normal University, Changsha, Hunan, People's Republic of China; The Research Institute for Children at Children's Hospital New Orleans, United States of America

## Abstract

Osteoporosis, the most prevalent metabolic bone disease among older people, increases risk for low trauma hip fractures (HF) that are associated with high morbidity and mortality. Hip bone size (BS) has been identified as one of the key measurable risk factors for HF. Although hip BS is highly genetically determined, genetic factors underlying the trait are still poorly defined. Here, we performed the first genome-wide association study (GWAS) of hip BS interrogating ∼380,000 SNPs on the Affymetrix platform in 1,000 homogeneous unrelated Caucasian subjects, including 501 females and 499 males. We identified a gene, *PLCL1* (phospholipase c-like 1), that had four SNPs associated with hip BS at, or approaching, a genome-wide significance level in our female subjects; the most significant SNP, rs7595412, achieved a *p* value of 3.72×10^−7^. The gene's importance to hip BS was replicated using the Illumina genotyping platform in an independent UK cohort containing 1,216 Caucasian females. Two SNPs of the *PLCL1* gene, rs892515 and rs9789480, surrounded by the four SNPs identified in our GWAS, achieved *p* values of 8.62×10^−3^ and 2.44×10^−3^, respectively, for association with hip BS. Imputation analyses on our GWAS and the UK samples further confirmed the replication signals; eight SNPs of the gene achieved combined imputed *p* values<10^−5^ in the two samples. The *PLCL1* gene's relevance to HF was also observed in a Chinese sample containing 403 females, including 266 with HF and 177 control subjects. A SNP of the *PLCL1* gene, rs3771362 that is only ∼0.6 kb apart from the most significant SNP detected in our GWAS (rs7595412), achieved a *p* value of 7.66×10^−3^ (odds ratio = 0.26) for association with HF. Additional biological support for the role of *PLCL1* in BS comes from previous demonstrations that the *PLCL1* protein inhibits IP3 (inositol 1,4,5-trisphosphate)-mediated calcium signaling, an important pathway regulating mechanical sensing of bone cells. Our findings suggest that *PLCL1* is a novel gene associated with variation in hip BS, and provide new insights into the pathogenesis of HF.

## Introduction

Osteoporosis is a serious public health problem in the elderly, leading to low trauma hip fractures (HF) that are associated with high morbidity and mortality. Hip bone size (BS) is one of the major parameters for hip bone quality and strength, and it has been shown that abnormal hip BS contributes significantly to the pathogenesis of HF [1], [2]. Consequently, hip BS has been identified as an important risk factor for HF.

Genetic factors play an important role in BS variation. One recent study demonstrated that the heritability of BS can reach as high as 75% [3], but specific genes underlying variation of BS, particularly hip BS, are still largely unknown. Only a limited number of association studies have been performed on hip BS to date, usually without full replication. They have implicated a few interesting candidate genes, such as *VDR* [4], *COL1A2* [5] and *CYP17* [6]. However each of these studies focused on genes with known significance in bone biology and, consequently, was not designed to identify potentially novel genes/regulatory mechanisms underlying hip BS.

A powerful strategy for identifying new genes associated with hip BS is genome-wide association studies (GWAS) that take advantage of the rapid development of high throughput SNP genotyping platforms combined with increasing knowledge of linkage equilibrium (LD) patterns in humans. The GWAS approach has demonstrated its great power to identify novel genes for human complex diseases/traits [7]–[11], using currently available high SNP density platforms that permit detection of culprit DNA changes within a narrow genomic region.

Here we conducted the first GWAS to search for novel genes underlying hip BS variation. Using Affymetrix 500 K arrays, we successfully genotyped and analyzed a total of ∼380,000 SNPs in 1,000 unrelated Caucasians, including 501 females and 499 males. We identified a gene, *PLCL1*, that was associated with hip BS at the genome-wide significance level in female subjects. The gene's association with hip BS was replicated in an independent cohort from UK containing 1,216 Caucasian women. More importantly, the gene's relevance to HF was observed in a Chinese sample containing 266 HF and 177 control subjects. (The subject characteristics of the cohorts used are detailed in Materials and Methods section and presented in Table 1.) Our findings strongly support the importance of the *PLCL1* gene to hip BS, and to the pathogenesis of HF.

**Table 1:** Basic characteristics of study subjects.

Traits	GWAS sample (N = 1,000)		UK replication sample (Female, N = 1,216)	Chinese HF sample (Female, N = 403)	
	*Female (N = 501)*	*Male (N = 499)*		*Case (N = 226)*	*Control (N = 177)*
Age (years)	50.1 (17.7)	50.5 (18.9)	48.7 (11.8)	72.1 (8.9)	67.7 (6.3)
Height (cm)	163.8 (6.5)	177.8 (7.0)	162.1 (6.2)	157.8 (4.7)	153.2 (6.7)
Weight (kg)	71.2 (15.9)	89.0 (14.9)	67.0 (12.7)	55.6 (11.2)	56.8 (10.1)
Hip BS (cm^2^)	34.2 (3.4)	44.7 (4.5)	33.6 (3.9)	−	−

Note: Presented as means (SD).

## Results

### GWAS findings

**Figure 1:**
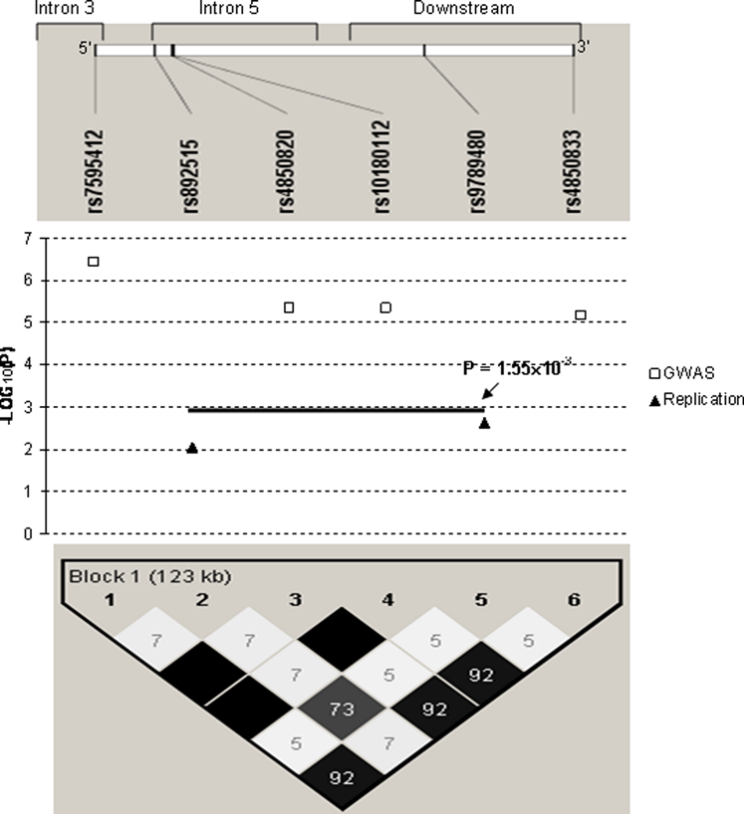
Association signals of *PLCL1* gene in GWAS and UK replication samples. Note: 1. This haplotype block map was constructed using the most recent SNP genotype data (HapMap Data Rel 23a/phaseII Mar 08, on NCBI B36 assembly, dbSNP b126) from HapMap (www.hapmap.org), showing pairwise LD in *r^2^*. 2. The black bar represents the association signal (*p* = 1.55×10^−3^) achieved in the UK replication sample for the haplotype block formed by the two SNPs, rs892515 and rs9789480.

We performed genome-wide genotypic association analyses for hip BS in our GWAS sample containing 1,000 subjects, but did not detect any SNP that passed the genome-wide significance threshold of 4.2×10^−7^ in the total sample. We then proceeded with gender-specific association analyses and identified a SNP, rs7595412, that achieved a genome-wide significant *p* value of 3.72×10^−7^ in our female subjects. Rs7595412 is in intron 3 of the *PLCL1* gene (Table 2 and Figure 1). Carriers of the major A allele of this SNP have on average ∼5 cm^2^ or ∼17% larger hip BS than non-carriers. In addition to the above SNP, three other SNPs in or near the *PLCL1* gene (rs4850820, rs10180112 and rs4850833) also achieved *p* values approaching genome-wide significance (*p* = 4.61×10^−6^, 4.73×10^−6^ and 7.04×10^−6^, respectively; Table 2). Of these three SNPs, two are in intron 5 and one is ∼72 kb downstream from the gene (Table 2 and Figure 1). Altogether these four SNPs span a region of ∼123 kb, and are in strong LD (Figure 1). Additional details about these four SNPs are presented in Table 2 and Figure 1.

**Table 2:** Information for SNPs of the *PLCL1* gene associated with hip BS or HF.

SNP Name	Position	Role	Allele^1^	MAF^2^	MAF^3^	P Value/OR^4^
*GWAS sample (association with hip BS)*						
rs7595412	Chr2: 198670488	Intron 3	A/G	0.112	0.117	3.72×10^−7^
rs4850820	Chr2: 198690456	Intron 5	G/C	0.124	0.117	4.61×10^−6^
rs10180112	Chr2: 198691103	Intron 5	G/C	0.125	0.117	4.73×10^−6^
rs4850833	Chr2: 198793906	downstream	G/A	0.139	0.125	7.04×10^−6^
*UK replication sample (association with hip BS)*						
rs892515	Chr2: 198686066	Intron 5	C/T	0.309	0.350	8.62×10^−3^
rs9789480	Chr2: 198755871	downstream	C/A	0.279	0.283	2.44×10^−3^
*Chinese HF sample (association with HF)*						
rs3771362	Chr2:198671076	Intron 3	C/T	0.214	0.267	7.66×10^−3^
						0.26 (0.09–0.75)

Note:

1 The second allele represents the minor allele of each locus.

2 Minor allele frequency calculated in our own Caucasian/Chinese sample.

3 Minor allele frequency reported for Caucasians in the public database of HapMap CEU, except for the MAF of rs3771362 that is from HapMap HCB for Chinese.

4 Odds ratio with 95% confidence interval for the SNP, rs3771362, is for the minor allele.

To give a more comprehensive presentation of our GWAS findings, we present in Appendix S1 the most significant 30 SNPs detected genome-wide in the total sample as well as in each gender subgroup.

### Replication of the PLCL1 gene's association with BS in the UK sample

In the UK replication sample, using a different genotyping platform, we also observed strong association signals for the *PLCL1* gene with hip BS. We compared the association signals of our GWAS with those achieved in the GWAS performed in the UK cohort. The SNPs/genes that achieved *p* values less than 0.01 in our GWAS were checked for *p* values achieved in the UK cohort, and vice versa for those SNPs/genes that achieved *p* values less than 0.01 in the UK cohort. As a result, the only gene that showed significant *in silico* replication signals is the *PLCL1* gene that had four SNPs associated with hip BS in our GWAS (*p*<10^−5^) (Table 2) and two SNPs associated with hip BS in the UK cohort (*p*<0.01) (Table 2).

A SNP in intron 5 of the *PLCL1* gene, rs892515, achieved a *p* value of 8.62×10^−3^ for association with hip BS in the UK sample. That SNP is located between the most significant and the 2^nd^ most significant SNPs of the *PLCL1* gene (i.e., rs7595412 and rs4850820, respectively) identified in our GWAS. The SNP rs892515 is separated from rs7595412 and rs4850820 by ∼15 kb and ∼4 kb, respectively (Table 2 and Figure 1). Another SNP, rs9789480, located ∼35 kb downstream from the *PLCL1* gene, achieved a *p* value of 2.44×10^−3^ in the UK sample. That SNP is positioned between two other interesting SNPs (i.e., rs10180112 and rs4850833) of the *PLCL1* gene identified in our GWAS (∼64 kb away from the former and ∼38 kb away from the latter SNP) (Table 2 and Figure 1). The haplotype formed by the two SNPs identified in the UK sample also achieved a *p* value of 1.55×10^−3^ for association with hip BS (Figure 1). Additional details about these two SNPs and their relative position in relation to the four SNPs identified in our GWAS is shown in Table 2 and Figure 1. As shown in Figure 1, rs892515 and rs9789480 have strong LD with each other, but weak LD with the four SNPs identified in our GWAS, even though these six SNPs are physically intermingled together.

### Imputation results on the region of the PLCL1 gene

**Figure 2:**
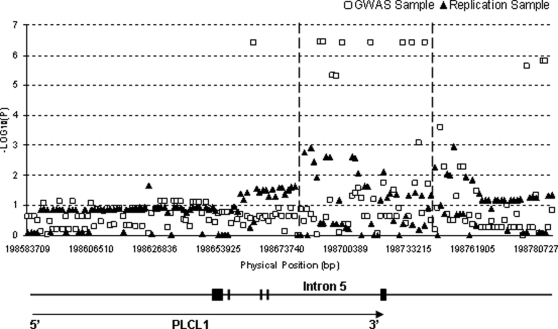
Imputation results for the *PLCL1* gene in our GWAS and UK replication samples.

As only ∼15% of SNPs overlap between the genotyping platform for our GWAS sample (i.e., Affymetrix) and that for the UK replication sample (i.e., Illumina), to better compare the association signals between the two samples, we imputed a common set of dense SNP markers covering the *PLCL1* gene and its vicinity. The imputed association signals for the whole span of the gene are plotted in Figure 2. According to the imputation results, eight SNP markers achieved *p* values less than 1×10^−6^, and five additional SNP markers achieved *p* values less than 1×10^−5^ in our GWAS sample. In the UK replication sample, more than ten SNP markers achieved *p* values less than 0.01. More importantly, the strongest association signals for both samples overlap nicely in a region covering ∼3/4 length of intron 5 and extending immediately downstream from the gene (shown in the Figure 2 as the region between the two dashed vertical lines).

**Figure 3:**
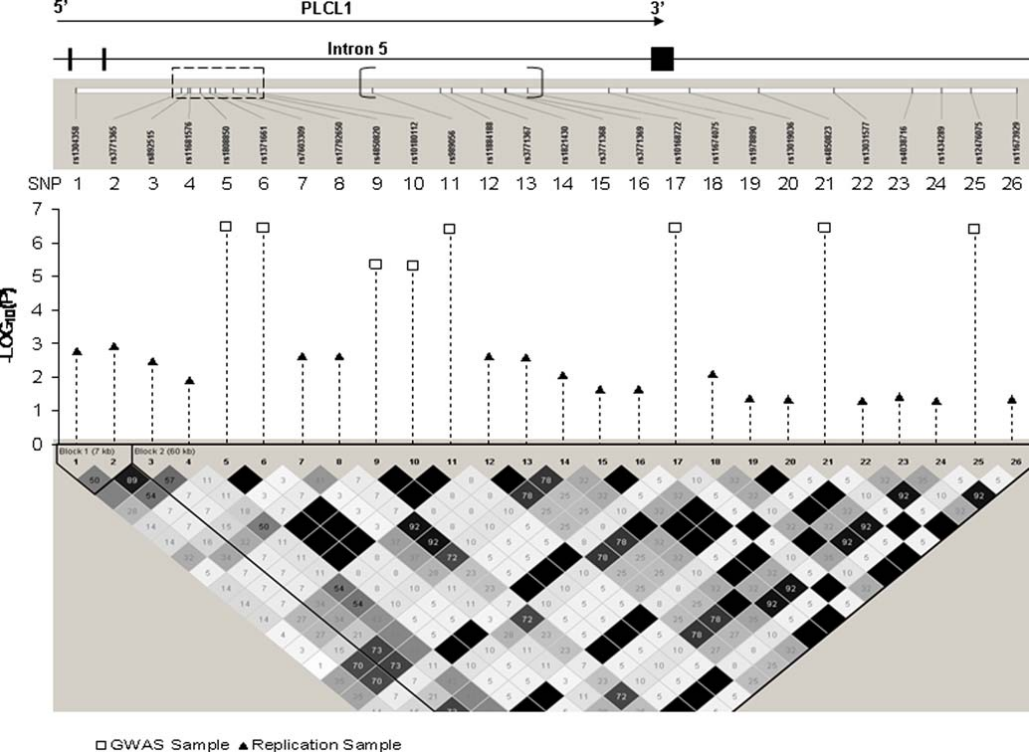
Association results imputed for intron 5 and the region immediately downstream from the *PLCL1* gene.

Figure 3 zooms in on the region between the two dashed lines, highlighted in Figure 2, to examine this region more closely; only those SNPs that achieved *p* values less than 1×10^−5^ in the GWAS sample, and those that achieved *p* values less than 0.05 in the UK sample, are shown. Detailed information for some of the plotted SNPs is presented in Table 3. A noticeable region in intron 5 with strong replication signals is highlighted in Figure 3 within the dashed rectangle (also designated “Region I” in Table 3). That region is less than 6 kb and contains nine significant SNP markers, according to imputation results. In that small region, two SNPs achieved genome-wide significant *p* values (*p*<4.2×10^−7^) and two achieved *p* values less than 1×10^−5^ in the GWAS sample; four other SNPs in this region achieved *p* values less than 0.01 in the UK sample. Another interesting region in intron 5 (demarcated in Figure 3 as inside brackets, and designated “Region II” in Table 3) is flanked by two SNPs, rs989056 and rs10168722, both of which achieved genome-wide significant *p* values (*p*<4.2×10^−7^) in the GWAS sample. In the UK sample, this region of ∼11 kb contained three SNPs with *p* values less than 0.01 and two additional SNPs with *p* values less than 0.05.

**Table 3:** Major imputation results for intron 5 of the *PLCL1* gene.

Region	GWAS Sample			Replication Sample		
	SNP	Position (kb)	P value	SNP	Position (kb)	P value
I	rs1808850	10.0	3.49×10^−7^	rs3771365	8.6	1.23×10^−3^
	rs1371661	10.7	3.67×10^−7^	rs892515	9.2	3.45×10^−3^
	rs4850820	13.6	4.61×10^−6^	rs11681576	9.3	1.21×10^−2^
	rs10180112	14.2	4.73×10^−6^	rs7603309	11.1	2.37×10^−3^
				rs17792650	12.4	2.47×10^−3^
II	rs989056	22.6	3.83×10^−7^	rs11884188	27.6	2.45×10^−3^
	rs10168722	33.9	3.77×10^−7^	rs3771367	28.4	2.61×10^−3^
				rs1821430	30.6	8.44×10^−3^
				rs3771368	32.3	2.32×10^−2^
				rs3771369	32.3	2.32×10^−2^

Note: Position (kb) is for the location of a SNP inside intron 5 of the *PLCL1* gene, calculated as the distance from the beginning of the intron.

Using Fisher's method [12], we combined the imputed *p* values achieved in the two samples. We found that eight SNPs of the *PLCL1* gene achieved combined imputed *p* values less than 10^−5^, another five SNPs achieved combined *p* values less than 10^−4^, and fifteen more SNPs achieved combined *p* values less than 0.01. These SNPs are mainly those as shown in Figure 3. Additional details of these SNPs are presented in Table 4.

**Table 4:** Combined imputed *p* values for SNPs of the *PLCL1* gene.

SNP	Position	Role	P value in GWAS	P value in UK sample	Combined P
rs7595412	198670488	Intron 3	3.74×10^−7^	3.53×10^−1^	2.22×10^−6^
rs1808850	198686906	Intron 5	3.49×10^−7^	3.95×10^−1^	2.31×10^−6^
rs1371661	198687636	Intron 5	3.67×10^−7^	3.97×10^−1^	2.44×10^−6^
rs4850823	198727690	downstream	3.77×10^−7^	4.10×10^−1^	2.58×10^−6^
rs10168722	198710808	Intron 5	3.77×10^−7^	4.12×10^−1^	2.59×10^−6^
rs989056	198699520	Intron 5	3.83×10^−7^	4.12×10^−1^	2.63×10^−6^
rs992603	198735269	downstream	3.77×10^−7^	4.36×10^−1^	2.73×10^−6^
rs12476075	198743194	downstream	3.85×10^−7^	4.53×10^−1^	2.89×10^−6^
rs4850447	198782135	downstream	1.51×10^−6^	8.13×10^−1^	1.80×10^−5^
rs2342753	198781579	downstream	1.58×10^−6^	8.07×10^−1^	1.85×10^−5^
rs10183584	198778210	downstream	2.40×10^−6^	7.50×10^−1^	2.56×10^−5^
rs4850820	198690456	Intron 5	4.61×10^−6^	4.06×10^−1^	2.66×10^−5^
rs10180112	198691103	Intron 5	4.73×10^−6^	4.06×10^−1^	2.72×10^−5^
rs3771367	198705284	Intron 5	4.04×10^−2^	2.61×10^−3^	1.07×10^−3^
rs11884188	198704457	Intron 5	4.84×10^−2^	2.45×10^−3^	1.19×10^−3^
rs11674075	198716751	Intron 5	1.79×10^−2^	7.77×10^−3^	1.37×10^−3^
rs17792650	198689347	Intron 5	6.78×10^−2^	2.47×10^−3^	1.62×10^−3^
rs1821430	198707471	Intron 5	2.69×10^−2^	8.44×10^−3^	2.14×10^−3^
rs3771365	198685530	Intron 5	1.89×10^−1^	1.23×10^−3^	2.17×10^−3^
rs9789480	198755871	downstream	3.14×10^−1^	1.08×10^−3^	3.06×10^−3^
rs1434288	198740743	downstream	8.23×10^−4^	6.30×10^−1^	4.44×10^−3^
rs1304358	198677828	Intron 5	3.29×10^−1^	1.70×10^−3^	4.75×10^−3^
rs7603309	198688008	Intron 5	2.50×10^−1^	2.37×10^−3^	5.00×10^−3^
rs4038716	198738934	downstream	1.88×10^−2^	3.88×10^−2^	5.99×10^−3^
rs892515	198686066	Intron 5	2.80×10^−1^	3.45×10^−3^	7.68×10^−3^
rs1119850	198751505	downstream	5.25×10^−3^	1.88×10^−1^	7.80×10^−3^
rs6434957	198757353	downstream	5.21×10^−3^	1.92×10^−1^	7.91×10^−3^
rs6735486	198757463	downstream	5.21×10^−3^	1.94×10^−1^	7.96×10^−3^

### PLCL1 gene's importance to HF

To further examine the *PLCL1* gene's importance to HF, we took advantage of a recently completed GWAS of HF in Chinese (containing 403 females and 297 males). We re-analyzed SNPs of the *PLCL1* gene, used for the previous GWAS, for association with HF in Chinese female subjects. The SNP that achieved the most significant *p* value in our GWAS in Caucasians (rs7595412) is not polymorphic in Chinese. However, a neighboring SNP, rs3771362, that is only ∼0.6 kb from rs7595412 achieved a *p* value of 7.66×10^−3^ for association with HF in the Chinese females, with an odds ratio (95% CI) of 0.26 (0.09–0.75) (Table 2). The SNP was not found to be associated with HF in the male subjects of the Chinese sample.

### PLCL1 gene's importance to other relevant phenotypes

In the female subjects of our GWAS cohort, we also analyzed the *PLCL1* gene's importance to other phenotypes relevant to osteoporosis, including hip and spine BMD, spine BS and height. The SNPs under analysis were the four SNPs of the gene that achieved the most significant results for association with hip BS in the female subjects of our GWAS cohort (Table 2).

We did not find significant association of the SNPs with hip and spine BMD and with height. However, we found marginally significant association of the SNPs with spine BS. The *p* values achieved by the SNPs (rs7595412, rs4850820, rs10180112 and rs4850833) are 0.048, 0.092, 0.092, and 0.056, respectively.

### BMI's effects on the PLCL1 gene's association with hip BS

Since osteoporosis incidence was found to be inversely associated with BMI [13], we also examined the influence of BMI on the *PLCL1* gene's association with hip BS. In our GWAS cohort (female subjects), BMI appears to have some effects on the *PLCL1* gene's association with hip BS. Adjusting hip BS with BMI in our GWAS sample increased the *p* values for the *PLCL1* gene's association with hip BS as shown in Table 2. After the adjustment, the *p* values for the SNPs rs7595412, rs4850820, rs10180112, and rs4850833 were increased to 1.15×10^−4^, 4.85×10^−4^, 4.85×10^−4^, and 3.03×10^−4^, respectively, as compared to the *p* values before the adjustment, which were 3.72×10^−7^, 4.61×10^−6^, 4.73×10^−6^, and 7.04×10^−6^, respectively. The results suggest that the *PLCL1* gene's association with hip BS may be partially mediated by BMI.

### Analyses for potential population stratification

To detect potential stratification of our GWAS sample, we analyzed our sample using software Structure 2.2 [14]. When 2,000 randomly selected un-linked markers were used to cluster our subjects, under all assigned values (i.e., 2, 3, or 4) for the assumed number of population strata, *k*, all subjects of the sample were tightly clustered together, suggesting no population stratification. The results are shown in Appendix S2.

We further tested our GWAS sample for population stratification using the genomic control method [15]. Based on genome-wide SNP information, we estimated the inflation factor (λ), a measure for population stratification. Ideally, for a homogeneous population with no stratification, the value of λ should be equal or near to 1.0. In our sample, the estimated λ value was 1.007, suggesting essentially no population stratification and further confirming the results achieved through the Structure 2.2 software.

We also analyzed the UK replication and Chinese HF samples for population stratification using the same approach described above for our GWAS sample, and achieved similar results. For the UK cohort, the subjects under study were those that remained after we excluded subjects who were not of European ancestry, according to analyses with Structure 2.2 [14]. The λ value for the UK sample was 1.02 according to the genomic control analysis [15]. For the Chinese HF sample, the Structure program showed that all subjects were clustered together as a homogeneous population, and a λ value of 1.02 was achieved through analysis with the genomic control method [15].

### Other analyses

Using the Q-Q plot, we examined the distribution of *p* values achieved in our GWAS for all of the ∼380,000 SNPs that were analyzed (Appendix S3). As shown in the plot, the observed *p* values match reasonably well with the expected *p* values over a wide range of values of [−LOG_10_(*p*)], which is from 0 to ∼4. Observed *p* values gradually depart from expected *p* values at the extreme tail, where [−LOG_10_(*p*)] is ≥∼4. The pattern suggests that our GWAS association findings were more likely due to true genetic variation than potential bias, such as genotyping errors.

Using the FASTSNP program [16], we analyzed the potential functions for the identified SNPs as shown in Table 2. According to the analyses, three SNPs identified in our GWAS sample, rs7959412, rs4850820 and rs10180112, and a SNP identified in the UK sample, rs892515, are potential intronic enhancers. A polymorphic A→G change at rs7959412 and a polymorphic G→C change at rs4850820 and rs10180112 may eliminate binding sites for the transcription factors (TFs), GATA-1 and CdxA, respectively, whereas a polymorphic C→T change at rs892515 may create a binding site for TFs, GATA-1 and XFD-1.

We compared the results of our GWAS with that of our most recently performed whole genome linkage study (WGLS) of BS [17]. In our previous WGLS the most significant region linked to hip BS was 8q24 that achieved a LOD score of 3.27 in the total sample and a LOD score of 3.01 in the female subgroup. Several genes in that region also achieved very strong association signals in our current GWAS. Two SNPs of the *ZFAT1* (zinc finger gene in autoimmune thyroid disease 1) gene, rs7462652 and rs7006328, achieved *p* values of 3.71×10^−5^ and 5.03×10^−5^, respectively, for association with hip BS in the total sample in our GWAS. In addition, three genes, *DEPDC6* (DEP domain containing 6), *EIF2C2* (eukaryotic translation initiation factor 2c, subunit 2) and *COLEC10* (collectin 10), each had a SNP (rs9297608, rs2977451 and rs16892015, respectively) associated with hip BS in the female subjects in our GWAS, with *p* values of 5.42×10^−5^, 5.86×10^−5^ and 1.05×10^−4^, respectively.

## Discussion

This study represents the first published GWAS for hip BS, an important risk factor for HF. Through this study we identified a novel gene, *PLCL1* that had four SNPs associated with hip BS at, or approaching, the genome-wide significance level in our female subjects. The gene's association with hip BS was replicated in an independent UK sample despite it using a different platform and selection of SNPs. Two SNPs in the UK sample, that are surrounded by the four interesting SNPs identified in our GWAS, were associated with hip BS with *p* values less than 0.01 (Figure 1). Imputation analyses of our GWAS and the UK samples showed a significant overlap of association signals in intron 5 of the *PLCL1* gene and immediately downstream from that gene (Figure 2). In particular, according to the imputation analyses, a narrow window of <6 kb in intron 5 contained four SNPs that achieved *p* values less than 1×10^−5^ (including two of genome-wide significance) in our GWAS sample, and four SNPs that achieved *p* values less than 0.01 in the UK sample, (Table 3 and Figure 3). Combining *p* values of the imputed SNPs in our GWAS and the UK samples, we found that eight SNPs of the *PLCL1* gene achieved combined *p* values less than 10^−5^, five additional SNPs achieved combined *p* values less than 10^−4^, and fifteen more SNPs achieved combined *p* values less than 0.01 (Table 4).

Importantly, we also demonstrated the relevance of *PLCL1* to HF. A SNP of this gene, rs3771362, that is only ∼0.6 kb away from the most significant SNP identified in our GWAS (rs7595412), achieved a *p* value of 7.66×10^−3^ and an odds ratio of 0.26 (95% CI: 0.09–0.75) for association with HF in an independent Chinese sample. *PLCL1* encodes an inositol 1,4,5-trisphosphate (IP3) binding protein that can inhibit IP3 mediated calcium signaling [18], an important pathway that regulates the response of bone cells to mechanical signals [19], [20]. Overall, our association findings, together with *PLCL1*'s potential functional relevance to bone mechanical sensing, provide strong evidence for the gene's importance for hip BS variation and the pathogenesis of HF.

In our GWAS sample, the *PLCL1* gene's association with hip BS was limited only to women, suggesting female-specificity of this gene's importance to osteoporosis. The female-specificity of the gene cannot be determined in the UK replication cohort since the cohort contains only women but no men [21]. However, analysis results in the Chinese HF cohort seem to support the female-specificity of the gene as the gene was associated with HF in females but not in males (data not shown). Our findings are consistent with previous studies suggesting that genetic control of bone parameters may be gender-specific [22]–[24].

In this study, replication of the *PLCL1* gene's association with hip BS in the UK sample was achieved “gene-wise” rather than “SNP-wise”. The most significant SNPs of the gene detected/imputed in our GWAS sample did not achieve nominally significant *p* values in the UK sample (Table 4), although the use of different genotyping platforms may be partly responsible. However, significant replication signals did show up in the UK sample in many other SNPs of the *PLCL1* gene, which are in neighborhood of those significant SNPs found in the GWAS sample, according to both the experimental data (Table 2 and Figure 1) and the imputation data (Table 3 and Figures 2 & 3).

BS can be measured as bone volume (cm^3^), bone area (cm^2^), or bone length (or diameter), and each of these measurements is legitimate and reflects various properties of bone. Areal BS measured by DXA, as adopted in this study, is a useful and reliable bone phenotype. Compared with other types of BS measurements, areal BS is relatively precise and involves less exposure to radiation during measurement [25], [26], enhancing its safety and feasibility for large-scale clinical investigation. Areal BS is also highly correlated with bone strength [27] and osteoporotic fractures [2], [28], [29], and this correlation is largely independent from BMD [2], [28], [29]. Consequently, genetic study of BS may provide a unique perspective to osteoporosis research, because current research is largely dominated by studies of BMD.

Population stratification and/or ethnic admixture can be an important source of spurious association in genetic association studies. These factors are unlikely to exist in our samples, however, and are therefore unlikely to interfere with our association results. The study cohort for our GWAS came from an apparently homogenous US mid-west white population, living in Omaha, Nebraska and its surrounding areas. The relative homogeneity of this population is largely due to the predominance of Caucasians as the major ethnic group in this area for many generations. According to the recent 2000 census, ethnic minorities made up only less than 3% of the entire population in the State of Nebraska. In addition, we found that the allelic frequencies of the significant SNPs in our GWAS sample are very similar to those reported in the typical and representative Caucasian samples used in the HapMap CEU (Table 2). Furthermore, using Structure 2.2 [14], we thoroughly analyzed study subjects used in our GWAS in order to detect potential sub-populations in the sample. In the analyses, all subjects tightly clustered together as a single group, suggesting no population substructure in our sample (Appendix S2). Calculated through the genomic control method [15], the measure for population stratification (λ) for our GWAS sample, was 1.007, suggesting essentially no stratification. Using the same approaches, we analyzed the UK replication sample and our Chinese HF sample and achieved similar results. For the above reasons, the association results, as detected in our study, are not likely to be plagued by spurious associations due to population admixture/stratification. Moreover the wide generalizability of the *PLCL1* gene's association makes this unlikely

In summary, we identified a novel gene, *PLCL1*, associated with hip BS through a GWAS. Our success in replicating the association of this gene with hip BS in a UK sample and the relevance of this gene to HF in a Chinese cohort, combined with the gene's functional implication in mechanical sensing by bone cells, makes it a strong candidate for regulation of hip BS and a potential key factor in the pathogenesis of HF. Apparent female specificity for the gene's association with hip BS supports gender-specific genetic basis of osteoporosis as suggested by previous studies [23], [24] and provides new insights into our understanding of differential HF risk between females and males. As a future direction of research, we will perform fine-mapping of the *PLCL1* gene in an enlarged independent Caucasian cohort to validate the gene's importance to hip BS and HF, including replicating the two SNPs that showed association with hip BS in the UK cohort (Table 2). In addition, focused molecular functional studies will be pursued to clarify the mechanisms by which this gene regulates hip BS variation and contributes to HF risk. The gene may also be related to bone growth in general – which also merits further work.

## Materials and Methods

### Subjects

The study was approved by the required Institutional Review Boards of all involved Institutions. Signed informed-consent documents were obtained from all study participants before they entered the study.

For the GWAS, a random sample containing 1,000 unrelated participants, including 501 women and 499 men, was identified from our established and expanding genetic repertoire currently containing more than 6000 subjects. All of the subjects were US Caucasians of European origin. Individuals with chronic diseases and conditions that might potentially affect bone metabolism were excluded. These diseases/conditions included chronic disorders involving vital organs (heart, lung, liver, kidney, brain), serious metabolic diseases (diabetes, hypo- and hyper-parathyroidism, hyperthyroidism, etc.), other skeletal diseases (paget disease, osteogenesis imperfecta, rheumatoid arthritis, etc.), chronic use of drugs affecting bone metabolism (hormone replacement therapy, corticosteroid therapy, anti-convulsant drugs), malnutrition conditions (such as chronic diarrhea, chronic ulcerative colitis, etc.), etc. Subjects taking anti-bone-resorptive or bone anabolic agents/drugs, such as bisphosphonates, were also excluded from this study. The purpose of the above exclusion criteria was to minimize the effects of environmental and therapeutic factors thought or known to influence the skeletal system and so improve power and ability to find genes.,

For our GWAS sample, areal BS values of total hip (proximal femur) were measured using dual energy X-ray absorptiometry (DXA) machines (Hologic Inc., Bedford, MA, USA) that were calibrated daily. The coefficients of variation (CV) of the DXA measurements for BS were about 1.94%. General relevant characteristics of the study subjects are presented in Table 1.

For replication of the *PLCL1* gene's association with hip BS, an independent sample containing 1,216 women of Caucasian European ancestry from the UK were used. The sample was selected from the TwinsUK cohort (www.twinsuk.ac.uk), a population-based sample of Britons previously shown to be representative of singleton populations, and the United Kingdom population, in general [30]. The areal BS of total hip site (proximal femur) was measured by DXA machines (Hologic Inc, Bedford, MA) using standard protocols. Basic characteristics of the UK study subjects are presented in Table 1.

To validate the relevance of the *PLCL1* gene to HF, we used a Chinese sample containing 403 female subjects including 226 with low trauma osteoporotic HF and 177 controls. All subjects were unrelated northern Chinese Han adults living in the city of Xi'an and its neighboring areas. Affected individuals with low trauma HF were recruited from the affiliated hospitals and associated clinics of Xi'an Jiaotong University. Inclusion criteria for HF patients were (i) age <80 years and onset age >55 years (all subjects were postmenopausal); (ii) HF caused by minimal or no trauma, usually due to falls from standing height or less; (iii) fracture site at the femoral neck or inter-trochanter regions; (iv) HF was identified/confirmed through diagnosis of orthopedic surgeons/radiologists according to radiological reports and x-rays. Patients with pathological fractures (e.g. due to tumors) and high-impact fractures (e.g. due to motor vehicle accidents) were excluded. Control subjects were enrolled through local advertisements. They were geography- and age-matched to the patients. All controls were older than 55 years, postmenopausal, and had no fracture history. Additional exclusion criteria for the control subjects were the same as those for our GWAS sample. The basic characteristics of the Chinese study subjects are shown in Table 1.

### Genotyping

#### GWAS sample

Genomic DNA was extracted from whole human blood using a commercial isolation kit (Gentra systems, Minneapolis, MN, USA) following protocols detailed in the kit. Genotyping with the Affymetrix Mapping 250 k Nsp and Affymetrix Mapping 250 k Sty arrays was performed in Vanderbilt Microarray Shared Resources (VMSR) (http://array.mc.vanderbilt.edu/) using the standard protocol recommended by Affymetrix. Genotyping calls were determined from the fluorescent intensities using the DM algorithm with a 0.33 *P*-value setting [31] as well as the B-RLMM algorithm [32]. DM calls were used for quality control while the B-RLMM calls were used for all subsequent data analysis. B-RLMM clustering was performed with 94 samples per cluster.

The final average BRLMM call rate across the entire sample reached the high level of 99.14%. However, out of the initial full-set of 500,568 SNPs, we discarded 32,961 SNPs with sample call rates <95%, another 36,965 SNPs with allele frequencies deviating from Hardy-Weinberg equilibrium (HWE) (*p*<0.001), and 51,323 SNPs with minor allele frequencies (MAF) <1%. Therefore, the final SNP set maintained in the subsequent analyses contained 379,319 SNPs, yielding an average marker spacing of ∼7.9 kb throughout the human genome.

#### UK replication sample

Subjects in the UK sample were genotyped with the Illumina Infinium assay across three genome-wide SNP sets, using fully compatible SNP arrays (Hap300 Duo, Hap300 and Hap550). Strict quality control criteria were applied, resulting in exclusion of 2,605 SNPs for violating Hardy-Weinberg equilibrium (*p*<10^−4^), 8,866 SNPs for low call rate (<90%), and 710 SNPs for low MAF (<0.01); 306,823 SNPs were retained for final association analyses.

#### Chinese HF sample

Subjects in the Chinese sample were genotyped with the Affymetrix Human Mapping 500 K array set (Affymetrix, Santa Clara, CA) following the same protocol used for our GWAS sample.

Quality control procedures were as follows. First, only samples with a minimum of 95% call rate were included; the final mean BRLMM call rate of the entire sample reached a high level of 99.02%. Second, out of the initial full set of 500,568 SNPs, we discarded: 1) SNPs with a call rate <90% in both cases and controls (*n* = 54,845); 2) those deviating from Hardy-Weinberg equilibrium (HWE) in controls (*p*<0.001, *n* = 22,002); 3) those having a minor allele frequency (MAF) <0.05 in the total sample (*n* = 142,188). Therefore, 281,533 SNPs were eventually available for subsequent association analyses.

### Statistical Analyses

#### GWAS sample

Parameters including age, age^2^, height and weight were tested for their association with hip BS. Significant (*p*≤0.05) terms were then included as covariates to adjust the raw BS values for subsequent analyses. The adjusted BS data, if not following normal distributions, were further subjected to BoxCox transformation into normal distribution. GWAS analyses were performed through genotypic association tests implemented in HelixTree 5.3.1 (Golden Helix, Bozeman, MT, USA). This commercial program incorporates the well known genetic analyses programs, FBAT and PBAT (http://www.biostat.harvard.edu/fbat/default.html), and the associated statistical methods [33].

The genome-wide significance threshold was set at *p* = 4.2×10^−7^, derived by Freimer and Sabatti [34] based on a gene-wise approach, and subsequently modified by Lencz et al. [35] taking into account a more accurate estimate of the total number of genes in the human genome.

#### UK replication sample

Raw hip BS values were adjusted through the same procedures, described above, for the GWAS sample. Association analyses were performed using the PLINK (QFAM) software package (version 1.01) (http://pngu.mgh.harvard.edu/purcell/plink/) accounting for family structure in the sample [36]. Some of the subjects are monozygotic twins, and for these sib-pairs, genotypic information for only one individual per pair was included in the analyses, since monozygotic twins share identical genetic information. Where a single dizygotic twin had missing data, or was excluded, the remaining sibling was treated as a singleton in the statistical analysis.

#### Chinese HF sample

Association analyses for the SNPs of the *PLCL1* gene were performed using the PLINK software package (version 1.02) [36]. Odds ratio for the SNP rs3771362 was calculated using logistic regression as implemented in Minitab package (Minitab Inc., State College, PA).

#### Quality control analyses

To detect population stratification in our GWAS sample, which may lead to spurious association results, we used the software Structure 2.2 (http://pritch.bsd.uchicago.edu/software.html) to investigate the potential substructure of our sample. The program uses a Markov chain Monte Carlo (MCMC) algorithm to cluster individuals into different cryptic sub-populations on the basis of multi-locus genotype data [14]. To ensure robustness of our results, we performed independent analyses under three assumed numbers for population strata (*k* = 2, 3, and 4), using 2,000 un-linked markers that were randomly selected across the entire genome. To confirm the results achieved through Structure 2.2, we further tested population stratification of our GWAS sample using a method of genomic control [15].

To investigate if the association findings in our GWAS were due to potential bias (e.g., genotyping error), we examined the distribution of *p* values for all ∼380,000 SNPs analyzed in our sample using the quantile-quantile (Q-Q) plot.

The program, Structure 2.2 [14], and the method of genomic control [15] were also applied to the UK replication sample and the Chinese HF sample to test for potential population stratification.

#### Other analyses

The SNPs of the *PLCL1* gene genotyped in our GWAS sample were largely different from that genotyped in the UK replication sample due to different genotyping platforms used for the two samples. In order to better compare association signals between the two samples at the region of this gene, we imputed a set of dense SNP markers common to both samples (HapMap data, NCBI Build 35) that cover the entire region based on the markers genotyped in the respective samples. To impute the genotypes of un-typed SNPs, we used the software IMPUTE [37] (http://www.stats.ox.ac.uk/marchini/software/gwas/impute.html). Based on the imputed genotypes, we performed single SNP association analyses using the software SNPTEST (http://www.stats.ox.ac.uk/marchini/software/gwas/snptest.html) to impute *p* values of un-typed SNPs. To quantify the overall significance of imputed association signals in both our GWAS and the UK samples, Fisher's method [12] was used to combine the individual *p* values imputed in each of the two samples.

To explore potential functions of the interesting SNPs identified in our GWAS and the UK replication samples, we used the FASTSNP (function analysis and selection tool for SNPs) program (http://fastsnp.ibms.sinica.edu.tw) that analyzes SNP functions based on up-to-date information extracted from 11 external bioinformatic databases at query time [16].

## Supporting Information

Appendix S1 The most significant 30 SNPs for hip BS detected in GWAS. Note: SNP A-1819962* does not have a dbSNP ID. The ID as shown in the table is an Affy ID.
(0.11 MB DOC)

Appendix S2 Results of analyses of potential population stratification for the GWAS sample using Structure 2.2. Note: As shown is output of the software Structure 2.2, which clustered our study subjects using 2,000 randomly selected unlinked markers under three assumed numbers of population strata, k = 2, 3, 4.
(0.04 MB TIF)

Appendix S3 Q-Q plots for the p values achieved in the GWAS
(0.05 MB TIF)
