# Correction: Identification of *PLCL1* Gene for Hip Bone Size Variation in Females in a Genome-Wide Association Study

**DOI:** 10.1371/annotation/951f0d10-0b78-4d6b-94c9-8ae6cc4178dd

**Published:** 2009-02-19

**Authors:** Yao-Zhong Liu, Scott G. Wilson, Liang Wang, Xiao-Gang Liu, Yan-Fang Guo, Jian Li, Han Yan, Panos Deloukas, Nicole Soranzo, Usha Chinappen-Horsley, Alessandra Cervino, Frances M. Williams, Dong-Hai Xiong, Yin-Ping Zhang, Tian-Bo Jin, Shawn Levy, Christopher J. Papasian, Betty M. Drees, James J. Hamilton, Robert R. Recker, Tim D. Spector, Hong-Wen Deng

There were several errors in the author names and affiliations. First, the 10th and 11th authors' names are misspelled. They should read, respectively: Usha Chinappen-Horsley and Alessandra Cervino.

Second, affiliation number 3 appears incorrectly. It should read: Department of Twin Research and Genetic Epidemiology, King’s College London, St Thomas’ Hospital, London, United Kingdom.

Third, several authors are shown with incorrect affiliations and should be corrected as follows: Panos Deloukas should be affiliated not with 3 but with 5 Wellcome Trust Sanger Institute, Wellcome Trust Genome Campus, Hinxton, United Kingdom. Usha Chinappen-Horsley, Alessandra Cervino, and Frances M. Williams should all be affiliated not with 5 but with 3.

