# Supplementary material for: Identification of PLCL1 Gene for Hip Bone Size Variation in Females in a Genome-Wide Association Study
Source: PLoS One. 2008 Sep 8;3(9):e3160. doi: 10.1371/journal.pone.0003160 (PMC2522269; doi:10.1371/journal.pone.0003160)
Supplement: Appendix S1 — The most significant 30 SNPs for hip BS detected in GWAS. Note: SNP A-1819962* does not have a dbSNP ID. The ID as shown in the table is an Affy ID. (0.11 MB DOC) [file pone.0003160.s001.doc]

**Appendix S1**

| Total sample | | | Female subgroup | | | Male subgroup | | |
| --- | --- | --- | --- | --- | --- | --- | --- | --- |
| dbSNP ID | p value | gene | dbSNP ID | p value | gene | dbSNP ID | p value | gene |
| rs8181826 | 1.65×10^-6^ | C1QTNF9 | rs7595412 | 3.72×10^-7^ | **PLCL1** | rs1075638 | 3.51×10^-6^ | PDK1 |
| rs2466398 | 1.66×10^-6^ | NPY | rs1286841 | 9.45×10^-7^ | TOP2B | rs893310 | 3.51×10^-6^ | LOC441177 |
| rs2466397 | 2.02×10^-6^ | NPY | rs2054897 | 1.24×10^-6^ | NGLY1 | rs3863747 | 4.81×10^-6^ | PLD5 |
| rs2521630 | 2.03×10^-6^ | NPY | rs6444443 | 1.31×10^-6^ | IL1RAP | rs390423 | 5.01×10^-6^ | FLJ32784 |
| rs2722381 | 2.31×10^-6^ | NPY | rs6547260 | 2.51×10^-6^ | CTNNA2 | rs12554091 | 5.89×10^-6^ | Unknown |
| rs4789205 | 3.22×10^-6^ | CASKIN2 | rs962207 | 3.94×10^-6^ | SLC9A2 | rs3863745 | 6.00×10^-6^ | PLD5 |
| rs17149209 | 3.30×10^-6^ | NPY | A-1819962* | 4.43×10^-6^ | LOC284402 | rs8070118 | 7.78×10^-6^ | HS3ST3A1 |
| rs11768564 | 3.53×10^-6^ | NPY | rs4850820 | 4.61×10^-6^ | **PLCL1** | rs673173 | 1.43×10^-5^ | FLJ46363 |
| rs11761673 | 3.75×10^-6^ | NPY | rs10180112 | 4.73×10^-6^ | **PLCL1** | rs282818 | 1.51×10^-5^ | Unknown |
| rs8075850 | 3.93×10^-6^ | CASKIN2 | rs714326 | 6.88×10^-6^ | KIAA1217 | rs16846743 | 2.51×10^-5^ | KCNH7 |
| rs2071239 | 7.11×10^-6^ | TCOF1 | rs4850833 | 7.04×10^-6^ | **PLCL1** | rs10878733 | 2.82×10^-5^ | DYRK2 |
| rs12554091 | 7.95×10^-6^ | Unknown | rs10894890 | 8.51×10^-6^ | Unknown | rs1433930 | 2.83×10^-5^ | PIK3C3 |
| rs3857716 | 8.55×10^-6^ | Unknown | rs6590773 | 9.30×10^-6^ | Unknown | rs4234723 | 2.89×10^-5^ | JAKMIP1 |
| rs6501805 | 1.25×10^-5^ | KIAA0195 | rs1487013 | 9.97×10^-6^ | Unknown | rs890448 | 3.06×10^-5^ | PPP3CA |
| rs196239 | 1.33×10^-5^ | INPP5F | rs7136015 | 1.10×10^-5^ | SYT1 | rs7846610 | 3.09×10^-5^ | CLN8 |
| rs474866 | 1.33×10^-5^ | CASP4 | rs7149043 | 1.25×10^-5^ | GSC | rs7135745 | 3.17×10^-5^ | FAM112B |
| rs893310 | 1.38×10^-5^ | LOC441177 | rs7577099 | 1.28×10^-5^ | LRRTM4 | rs7304798 | 3.17×10^-5^ | FAM112B |
| rs11953588 | 1.49×10^-5^ | MSX2 | rs7784081 | 1.39×10^-5^ | OSBPL3 | rs3798867 | 3.23×10^-5^ | SNAP91 |
| rs6461794 | 1.81×10^-5^ | NPY | rs4907093 | 1.42×10^-5^ | SSX2IP | rs11884875 | 3.55×10^-5^ | KCNH7 |
| rs9384218 | 2.05×10^-5^ | CNKSR3 | rs2428776 | 1.44×10^-5^ | CADPS2 | rs9927029 | 3.56×10^-5^ | ZFP1 |
| rs488137 | 2.56×10^-5^ | HNT | rs3857116 | 1.44×10^-5^ | Unknown | rs188848 | 3.66×10^-5^ | DYNC1I1 |
| rs7497765 | 2.87×10^-5^ | SLCO3A1 | rs2230906 | 1.49×10^-5^ | GCNT2 | rs263101 | 3.69×10^-5^ | LDLRAD3 |
| rs4832201 | 2.96×10^-5^ | ATOH8 | rs11022298 | 1.64×10^-5^ | MICALCL | rs6986249 | 3.95×10^-5^ | FKSG2 |
| rs6819993 | 3.11×10^-5^ | PRSS12 | rs17149209 | 1.67×10^-5^ | NPY | rs10831575 | 3.96×10^-5^ | GALNTL4 |
| rs2345408 | 3.15×10^-5^ | FLJ36144 | rs4502543 | 1.67×10^-5^ | Unknown | rs16892988 | 4.05×10^-5^ | PARK2 |
| rs487303 | 3.16×10^-5^ | HNT | rs17036521 | 1.73×10^-5^ | Unknown | rs4369056 | 4.07×10^-5^ | IKBKAP |
| rs1334103 | 3.26×10^-5^ | C20orf85 | rs11768564 | 1.77×10^-5^ | NPY | rs962115 | 4.16×10^-5^ | IGSF11 |
| rs2054897 | 3.33×10^-5^ | NGLY1 | rs634413 | 1.92×10^-5^ | LOC440131 | rs12741740 | 4.29×10^-5^ | FLJ32784 |
| rs1286841 | 3.69×10^-5^ | TOP2B | rs11761673 | 1.93×10^-5^ | NPY | rs1148283 | 4.34×10^-5^ | ZNF248 |
| rs7462652 | 3.71×10^-5^ | ZFAT1 | rs10518182 | 1.94×10^-5^ | MRPL1 | rs7520320 | 4.35×10^-5^ | SYT6 |
