# Supplementary figures and images for: Identification of PLCL1 Gene for Hip Bone Size Variation in Females in a Genome-Wide Association Study
Source: PLoS One. 2008 Sep 8;3(9):e3160. doi: 10.1371/journal.pone.0003160 (PMC2522269; doi:10.1371/journal.pone.0003160)

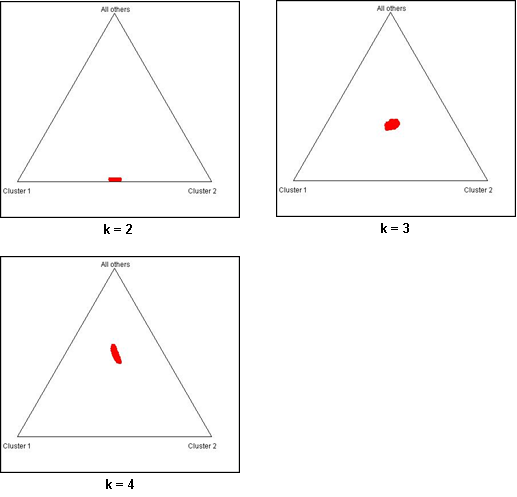

Supplement: Appendix S2 — Results of analyses of potential population stratification for the GWAS sample using Structure 2.2. Note: As shown is output of the software Structure 2.2, which clustered our study subjects using 2,000 randomly selected unlinked markers under three assumed numbers of population strata, k = 2, 3, 4. (0.04 MB TIF) [file pone.0003160.s002.tif]

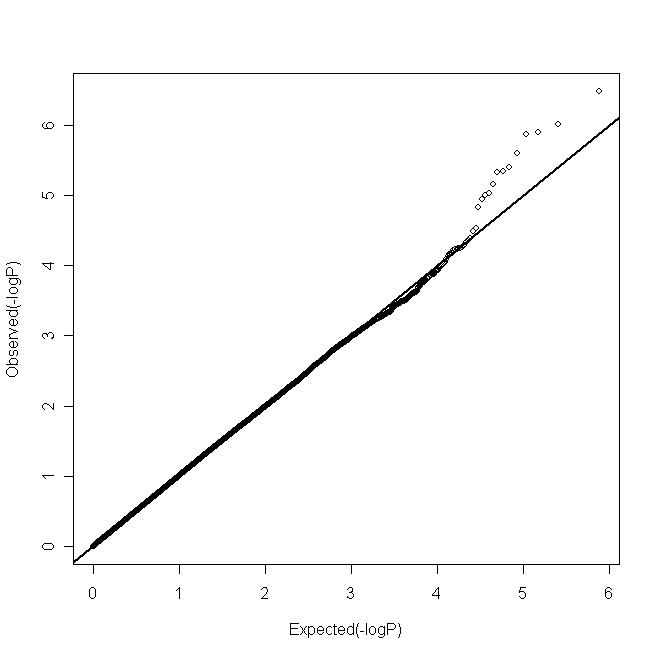

Supplement: Appendix S3 — Q-Q plots for the p values achieved in the GWAS (0.05 MB TIF) [file pone.0003160.s003.tif]
